# Random Parameter Sampling of a Generic Three-Tier MAPK Cascade Model Reveals Major Factors Affecting Its Versatile Dynamics

**DOI:** 10.1371/journal.pone.0054441

**Published:** 2013-01-24

**Authors:** Zhongxing Mai, Haiyan Liu

**Affiliations:** School of Life Sciences and Hefei National Laboratory for Physical Sciences at the Microscale, University of Science and Technology of China, Hefei, Anhui, People's Republic of China; SUNY Downstate MC, United States of America

## Abstract

The mitogen-activated protein kinase (MAPK) pathway is considered to be a central block in many biological signaling networks. Despite the common core cascade structure, the activation of MAPK in different biological systems can exhibit different types of dynamic behaviors. Computer modeling may help to reveal the mechanisms underlying such variations. However, so far most computational models of the MAPK cascade have been system-specific, or to reflect a particular type among the wide spectrum of possible dynamics. To obtain a general and integrated view of the relationship between the dynamics of MAPK activation and the structures and parameters of the MAPK cascade, we constructed a generic model by comparing previous models covering different specific biological systems. We assumed that reliable qualitative results could be predicted through a qualitative model with pseudo parameters. We used randomly sampled parameters instead of a specific set of “best-fit” parameters to avoid biases towards any particular systems. A range of dynamics behaviors for MAPK activation, including ultrasensitivity, bistability, transient activation and oscillation, were successfully predicted by the generic model. The results indicated that the steady state dynamics (ultrasensitivity and bistability) was jointly determined by the three-tiered structure of the MAPK cascade and the competitive substrate binding in the dual-phosphorylation processes of the central components, while the temporal dynamics (transient activation and oscillation) was mainly affected by the upstream signaling pathway and feedbacks. Moreover, MAPK kinase (MAPKK) played more important roles than the other two components in determining the dynamics of MAPK activation. We hypothesize that this is an important and advantageous property for the regulation and for the functional diversity of MAPK pathways in real cells. Finally, to assist developing generic models for signaling motifs through model comparisons, we proposed a reaction-based database to make the model data more flexible and interoperable.

## Introduction

The mitogen-activated protein kinase (MAPK) cascade is a central block in many cell signaling networks. This cascade presents widely in cell signaling pathways associated with proliferation, differentiation and apoptosis [1]–[3]. MAPKs are a family of cellular kinases. When activated, they activate a number of downstream substrates that regulate transcription and translation [4], [5]. The activation of a MAPK generally involves two sub-pathways: an upstream sub-pathway (e.g. the growth factor pathway [6] or the tumor necrosis factor pathway [7]) that lead to the activation of a MAPKK kinase (MAPKKK), and the central MAPK cascade that lead to the activation of a MAPK.

The central MAPK cascade has the following three-tiered core structure [8]:
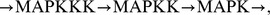
wherein MAPKKK (or MAP3K) is the entry component. Its activation triggers the cascade [9]. The active MAPKKK activates its cognate downstream MAPKK (or MAP2K) by phosphorylating the latter on two serine residues [10]. The phosphorylated MAPKK subsequently activates its downstream MAPK, also through dual phosphorylation [11]. The activation status of MAPK is the eventual output of this cascade.

Despite the apparently common and simple structure of the above central cascade, the activation of MAPK in different cell signaling pathways may display dynamics of diverse types, including ultrasensitivity, bistability, transient activation and oscillation [12]–[17]. With ultrasensitivity, the level of MAPK activation can vary dramatically upon small changes in an upstream stimulus, namely, a small increase of the stimulus from below to above a threshold can cause the activation of MAPK to increase rapidly from a low level to full activation [12], leading to an all-or-none type of response [13]. With bistability, MAPK activity can be maintained at two different steady state levels under certain conditions, e.g., with the strength of the stimulus within a certain range [14], [15]. As the actual state can be dependent on the history of the system, bistability can lead to irreversible responses. With transient activation, the level of MAPK activation may increase rapidly in initial response to the turning on of a stimulus, but may later drop back to a lower level even though the stimulus is maintained [16]. In certain systems, the level of MAPK activation may also oscillate with time after a stimulus is turned on [17].

It has been suggested that the different types of dynamics for MAPK activations may have implications for biological functions. For examples, ultrasensitivity and bistability are important for the MAPK cascade to function as a switch, while transient activation may serve the purpose of signal selection in some cases [18]. In addition, the oscillation dynamics of MAPK activation may be related to periodic gene expression and biological clocks [19]. It is thus interesting to understand how the different types of dynamics may emerge from the same core cascade structure. For examples, among the many structure and parameter components that comprise the cascade, what are the determining factors? And how are the different factors coupled to each other?

In general, computer models can be employed to address these questions. A computer model can be developed for a specific MAPK system. However, such a model is usually associated with a large extent of uncertainties in terms of structures and parameters because of limited knowledge of the real network. This problem may be partially addressed by limiting the network size, estimating parameters based on experimental data [20], [21] or referring to online databases [22]. Alternatively, computer models can aim at understanding properties of generic network structures rather than of specific systems. The versatile dynamics of the central MAPK cascade provide a suitable target for such studies. Unlike the development of models for specific systems, the focus in such modeling is no longer on system-specific quantitative parameters and predictions, but on the qualitative relationships between properties/functions of the system and possible variations of the system in the structure and/or parameter space (e.g., sensitivity analysis [23]). With computer modeling, such variations can be explored systematically, subjecting to known biological constraints to maintain maximum biological relevance of the results.

Previously, a number of system-specific computer models have been developed to analyze the dynamics of MAPK cascades in different biological systems. Various types of dynamics have been predicted. In this report, we perform a systematic analysis of possible dynamic behaviors of the central MAPK cascade that had been investigated in different previous models based on one generic model. In order to maintain generality without losing biological relevance, the generic model has been constructed by comparing/unifying 13 previous models of different specific MAPK cascades. This has been enabled using a data structure/database defined to facilitate the comparisons of mathematical models. The generic model is analyzed by varying the model structure using the structures of the previous system-specific models as guides, and by systematic sampling in its parameter space. The results provided a general picture of how the diverse types of dynamics may emerge from the same core MAPK cascade.

## Materials and Methods

### 2.1 A reaction-based database for analyzing and comparing mathematical models of biological networks

First, we semi-automated the procedure of performing objective and well-defined comparisons (and possibly unifications) of biological models. This is facilitated by defining a unified data structure to store different mathematical models of various biological networks into one single database, allowing the models to be searched or queried in various ways.

There have been a number of online databases for storing pre-defined models of biological networks, such as DOQCS (http://doqcs.ncbs.res.in) [24], SigPath (http://icb.med.cornell.edu/crt/SigPath/index.xml) [25] and BioModels (http://www.ebi.ac.uk/biomodels-main) [26]. However, we found it difficult to compare or unify models as stored in these databases. The reason is that the data structures used in these databases are model-based, i.e., one complete model corresponds to one unit record stored in a special format such as SBML [27]. With this type of data structures, automatic comparisons of names and values of model components (e.g., species, parameters, and the expressions for kinetic equations) are not meaningful even if some of the components in different models actually refer to the same or similar biological entities or processes. In addition, the formats storing the models have been designed for processing using computer programs but not for human comprehension, so manual comparisons of models are also difficult.

To resolve this issue, we defined a new reaction-based data structure, changing the unit data records from models into reactions. The expressions of chemical species and reactions in different models are unified or associated with each other if they refer to the same or similar biological entities/processes. Such a data structure improves the flexibility and interoperability of the stored data composing different models while preserving the convenience for the exchange and reuse of a certain model. For example, for any given model or reaction, related models as well as related reactions in current or other models in the database can be easily queried. Thus different models for the same or similar biological processes can be compared. Details of this data structure are provided in the supplementary material as Table S1 and Text S1.

**Figure 1:**
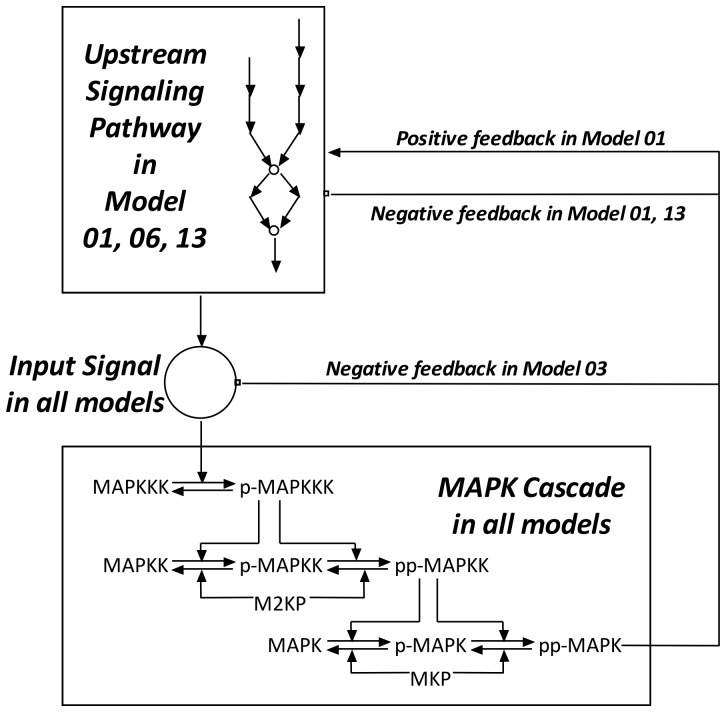
A schematic drawing summarizing the topologies of the MAPK activation network studied by previous models. The numbers are numeric IDs of the models as they are referred in the text and Table 1. The prefix ‘p-’ means phosphorylated and ‘pp-’ means dual-phosphorylated.

Assisted by this data structure, 13 previous models of MAPK cascade in different specific biosystems are compared (see Figure 1, Table 1 and also the results section). Based on the comparisons, a generic MAPK cascade model was defined.

**Table 1:** A summary of previously reported MAPK models.

ID	Specific Biosystem	Network Topology		Dynamic Behaviors			
		Upstream pathway	Feedback	Ultrasensitivity	Transient Activation	Oscillation	Bistability
1	CHO	EGF with 2 receptors	Positive and Negative	Not considered	Yes	No	Not considered
2	Xenopus oocyte	None	None	Yes	Not considered	Not considered	Not considered
3	None	None	Negative	Yes	No	Yes	Not considered
4	None	None	None	Yes	No	No	Not considered
5	None	None	None	Yes	No	No	Not considered
6	HeLa	EGF	None	Not considered	No	No	Not considered
7	None	None	None	Not considered	Not considered	Not considered	Yes
8	None	None	None	Not considered	Not considered	Not considered	Yes
9	None	None	None	Not considered	Not considered	Not considered	Yes
10	None	None	None	Not considered	Not considered	Not considered	Yes
11	None	None	None	Not considered	Not considered	Not considered	Yes
12	None	None	None	Not considered	Not considered	Not considered	Yes
13	PC12	EGF and NGF	Negative	Yes	Yes	No	Not considered

Field description:

**ID**: Numeric IDs of the models as they are referred in the text.

**Specific Biosystem**: Biosystem mapped by the model.

**Network Topology**: Upstream pathway or feedback involved in the model.

**Dynamic Behaviors**: MAPK dynamic behaviors emerged in the model.

#### 2.2.1 The framework of the model

**Figure 2:**
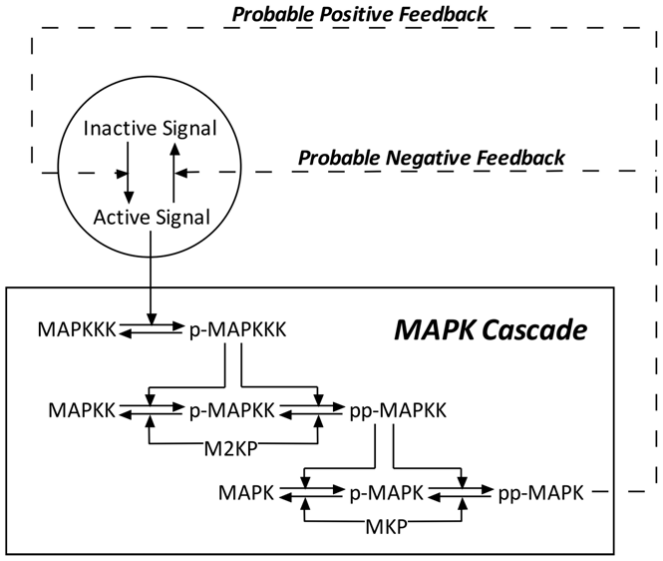
The topology of the generic model for MAPK activation. The presence or absence of the two probable feedbacks depends on the chosen kinetic parameters. In choosing the parameters, we applied the constraints that a particular parameter set can lead to either no feedback or the presence of only one of the feedbacks, but not the simultaneous presence of both feedbacks. In the Figure, the prefix p- means phosphorylated and pp- means dual-phosphorylated.

A diagram of the model is shown in Figure 2. Detailed upstream pathways have been excluded for simplicity as well as for generality. The activation of MAPKKK was treated as a single reaction step with a rate controlled by the strength of an input stimulus (see below). The activation of MAPKK and of MAPK was treated as two-step, enzyme-catalyzed dual-phosphorylation reactions. The inactivation of MAPKKK was treated as a first order reaction, while the inactivation of MAPKK and of MAPK was treated as two-step, enzyme-catalyzed dephosphorylation reactions involving phosphatases MAPKK phosphatase (M2KP) and MAPK phosphatase (MKP), respectively.

In our generic model, the input signal has been treated as a pseudo molecular species in a dynamic equilibrium between its active and inactive form. The active form was considered as the stimulus for MAPKKK activation. Feedback loops, if considered, have been modeled as effects of downstream species on the activating or inactivating rates of the input.

#### 2.2.2 Kinetic equations

For investigating the potential enzyme-substrate competitive binding in the enzyme-catalyzed reactions, we divided each enzyme-catalyzed reaction into a binding step and a catalytic step instead of using the Michaelis-Menten equation. The kinetic equations in our models has the following general form,
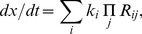
(1)where *k_i_* is the kinetic rate of the *ith* reaction involving *x*, *R_ij_* is the concentration of the *jth* reactant in reaction *i*. The complete model contains 20 (total) concentrations and 28 kinetic rates as parameters. Further details of the kinetic equations are given in Supplementary material.

#### 2.2.3 Sampling in the parameter space

For generality, the parameters in our model are assigned only relative values. We chose the total concentration of MAPKKK ([MAPKKK]_total_) and the rate of activation of MAPKKK as the basic units for concentrations and kinetic rates (and consequently, time), respectively. The values of these two parameters have been fixed to 1, and the values of the other parameter have been varied either systematically or through random sampling.

The space of initial concentrations has been systematically explored in the following manner. The total concentrations of MAPKK ([MAPKK]_total_) and MAPK ([MAPK]_total_) have been sampled as 0.2, 1.0 or 5.0 times of [MAPKKK]_total_. The total concentrations of phosphatases ([M2KP]_total_ and [MKP]_total_) have been sampled as 0.1 or 1.0 times of the total concentrations of their respective substrates. We considered the concentrations of products to be zero at the beginning of simulations, so there were only 4 initial concentrations ([MAPKK], [MAPK], [M2KP] and [MKP]) whose combinations need to be explored. The total number of combinations is 3*3*2*2 = 36.

All of the 28 kinetic rates need to be explored in combinations, which are too many to enumerate (for example, there will be 2^28^ combinations even if each kinetic rate can assume only two possible values). Thus the set of kinetic rates have been sampled randomly. After trying different sample sizes, we considered 2,000 sets of kinetic rates to be a sufficient size (based on the ratios of responsive models, see below) and the corresponding computational cost was still affordable. We note that the presence or absence of the feedbacks in Figure 2 was implemented by constraining the respective rate constants to zero or non-zero values, respectively. Finally, our simulations have been performed with 72,000 parameter combinations (36 sets of systematically varied initial concentrations combined with 2,000 sets of randomly selected kinetic rates). More details of parameter sampling are given in Supplementary material.

For each combination of parameters, we solved the set of ordinary differential equations in (1) using Matlab to obtain the time evolution of the system for a sufficiently long time. The model can be downloaded from https://senselab.med.yale.edu/ModelDB/showmodel.asp?model=146024.

## Results

### 3.1 Comparisons of previous models

We entered 13 MAPK cascade models from 7 references into our reaction-based database. Table 1 listed for each model the biosystem, the network topology and the types of dynamics predicted by the model. The network topologies of the MAPK cascade described by the 13 models are summarized in a single diagram in Figure 1. More detailed information regarding these models and their predicted dynamics will be described in Discussion.

### 3.2 Numerical simulations of the generic model

Time trajectories were obtained by solving the ordinary differential equations in (1). Each trajectory was characterized using well-defined numeric indicators. Criteria for responsive, ultrasensitive or bistable models were also defined, respectively, based on these indicators. With these criteria, respective statistics of 2000 sets of randomly sampled kinetic parameters were obtained for each of the 36 sets of different initial concentrations. The results are presented in Table 2. The numeric indicators, criteria and statistics are described below.

**Table 2:** Concentration vectors and statistics of MAPK activation.

ID	MAPKK^a^	MAPK^a^	M2KP^a^	MKP^a^	*r_EA_* (%)^b^	*r_SU_* (%)^b^	*r_BI_* (%)^b^
1	0.2	0.2	0.02	0.02	83.95	79.75	60.51
2	0.2	0.2	0.02	0.2	53.15	65.00	43.84
3	0.2	0.2	0.2	0.02	69.90	56.22	40.13
4	0.2	0.2	0.2	0.2	39.95	37.55	21.65
5	0.2	1	0.02	0.1	67.20	68.38	51.93
6	0.2	1	0.02	1	18.65	53.62	42.63
7	0.2	1	0.2	0.1	52.55	41.29	27.40
8	0.2	1	0.2	1	12.00	27.50	20.42
9	0.2	5	0.02	0.5	19.10	50.00	43.72
10	0.2	5	0.02	5	0.15	33.33	33.33
11	0.2	5	0.2	0.5	12.85	28.79	19.84
12	0.2	5	0.2	5	0.05	0.00	0.00
13	1	0.2	0.1	0.02	95.10	81.18	53.94
14	1	0.2	0.1	0.2	78.15	61.23	32.37
15	1	0.2	1	0.02	69.70	49.93	35.72
16	1	0.2	1	0.2	45.65	29.57	15.12
17	1	1	0.1	0.1	89.95	67.43	40.69
18	1	1	0.1	1	47.90	46.24	28.39
19	1	1	1	0.1	57.40	37.02	21.69
20	1	1	1	1	22.60	19.47	11.28
21	1	5	0.1	0.5	70.15	54.38	35.00
22	1	5	0.1	5	8.30	34.94	30.72
23	1	5	1	0.5	36.30	28.51	18.73
24	1	5	1	5	3.40	11.76	16.18
25	5	0.2	0.5	0.02	94.90	77.13	48.74
26	5	0.2	0.5	0.2	84.65	55.46	25.16
27	5	0.2	5	0.02	44.00	40.34	30.00
28	5	0.2	5	0.2	25.15	22.47	9.15
29	5	1	0.5	0.1	91.65	65.58	34.21
30	5	1	0.5	1	62.45	42.35	21.22
31	5	1	5	0.1	34.00	28.97	14.71
32	5	1	5	1	12.40	16.53	6.05
33	5	5	0.5	0.5	84.60	54.96	29.55
34	5	5	0.5	5	28.95	29.36	17.44
35	5	5	5	0.5	23.00	21.96	10.43
36	5	5	5	5	3.65	9.59	4.11

a Total concentrations of components.

b Percentages of parameter sets that can induce effective activation (*O*_max_>0.1, *r_EA_*), significant ultrasensitivity (*Gradient*>1.0, *r_SU_*) and bistability (*Bistability*>1.5, *r_BI_*), respectively.

#### 3.2.1 The output or strength of MAPK activation and responsive models

The ratio between the concentration of dual-phosphorylated MAPK ([pp-MAPK]) and [MAPK]_total_ was considered as the strength of MAPK activation, or output,
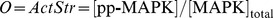
(2)


The models in which the maximum activation strength of MAPK in the simulated time course, *O*_max_, is larger than 0.1 have been considered as “responsive”. Since it makes no sense to investigate the dynamics of a model that could not produce effective activation, we are only concerned with parameters that lead to responsive models. The fraction of responsive parameter combinations for each of the 36 sets of initial concentrations, or the effective activating ratio, has been calculated as
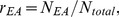
(3)where 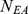 represented the number of parameter sets giving responsive models, and 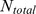 represented the total number of parameter sets.

#### 3.2.2 The effective gradient of output with respect to signal strength and ultrasensitive models

This effective gradient was used as an indicator for ultrasensitivity. Its value reflect how rapidly the level of MAPK activation changes with the stimulus during the “turning on” process,
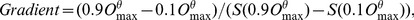
(4)where 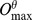 is the maximum (saturated) steady state output obtained from the simulations. *S*(x) is the stimulus strength that induces steady state output x.

Models with *Gradient* larger than 1.0 were considered as significantly ultrasensitive. For each of the 36 combinations of initial concentrations, we defined the ratio of significantly ultrasensitive models as
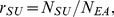
(5)where 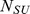 represented the number of parameters inducing significant ultrasensitivity, while 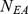 represented the number of parameter sets yielding responsive models.

#### 3.2.3 Indicators of bistability

We defined the indicators of bistability by the following steps. For each model (noted as *M*_i_) constructed by randomly selecting an initial concentration vector (noted as *ICV*_i_) and a kinetic rate vector (noted as *KRV*_i_), a set of steady-state concentration vectors *SCV* is obtained by simulations under a series of stimuli strength *S* that cover a range from 0 to a saturating strength. Then the *ICV*_i_ was replaced by the maximum-response steady-state concentration vector *SCV*_max_ and a new set of steady-state concentrations *SCV'* was obtain by repeating the simulations with the same series of *S*. To judge whether *M*_i_ was bistable, the respective output *SCV* and *SCV'* under a given stimulus strength (the check point) were compared,
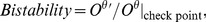
(6)where 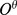 is the output value of *SCV*, and 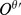 is the output value of *SCV'*. The check point was chosen as *S*(0.1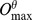). By this choice we only considered bistability that covered a significant range of input strength.

Theoretically, the value of *Bistability* should be in the range of (1.0, 10.0) under this definition, with a mono-stable model having a *Bistability* equal to 1.0 and a bistable model having a *Bistability* larger than 1.0. To account for the inevitable effects of numerical inaccuracies, we defined a model to be bistable if it showed a *Bistability* larger than 1.5. For each of the 36 combinations of initial concentrations, we defined the ratio of bistable models as
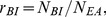
(7)where 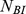 represents the number of parameters inducing bistability, and 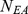 represents the number of parameter sets giving responsive models.

Moreover, we defined the models in which the 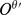 was very close to 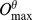 (i.e., *Bistability*>9.5) as absolutely bistable. Table 3 shows the numbers of parameters leading to absolute bistability with different initial concentrations. To consider the effects of feedbacks on bistability, models with and without certain feedback connections were compared in this table.

**Table 3:** Number of (absolutely) bistable models obtained with different concentration vectors under different network topologies.

Concentration Vectors	Number of (absolutely) bistable models under different network topologies		
	No feedback	Positive Feedback	Negative Feedback
1	327 (106)	348 (78)	341 (81)
2	153 (28)	156 (39)	157 (33)
3	185 (42)	204 (42)	172 (40)
4	51 (11)	75 (18)	47 (11)
5	232 (51)	242 (48)	224 (58)
6	57 (8)	55 (13)	47 (9)
7	93 (24)	117 (27)	78 (20)
8	15 (3)	17 (3)	17 (3)
9	61 (16)	52 (12)	54 (10)
10	0 (0)	1 (1)	1 (0)
11	16 (0)	18 (5)	17 (4)
12	0 (0)	0 (0)	0 (0)
13	337 (75)	353 (70)	336 (76)
14	168 (45)	172 (33)	166 (38)
15	162 (27)	191 (34)	145 (26)
16	43 (8)	55 (10)	40 (6)
17	242 (48)	272 (55)	218 (56)
18	94 (20)	101 (20)	77 (16)
19	82 (14)	109 (9)	58 (16)
20	13 (2)	23 (4)	15 (3)
21	171 (36)	205 (41)	115 (33)
22	14 (4)	23 (5)	14 (2)
23	45 (5)	67 (6)	24 (1)
24	5 (1)	4 (0)	2 (0)
25	308 (17)	322 (19)	295 (24)
26	149 (9)	151 (8)	126 (8)
27	74 (9)	108 (12)	82 (11)
28	9 (2)	21 (3)	16 (5)
29	224 (44)	240 (51)	163 (46)
30	93 (22)	103 (17)	69 (17)
31	25 (9)	47 (5)	28 (4)
32	4 (2)	8 (0)	3 (0)
33	176 (36)	210 (32)	114 (25)
34	42 (8)	34 (8)	25 (7)
35	10 (0)	29 (1)	9 (0)
36	2 (0)	1 (0)	0 (0)
Total	3682 (732)	4134 (729)	3295 (689)

#### 3.2.4 Simulation results

Over 45% of total 72,000 models (32,906) were found to be responsive under the definition of 3.2.1. Steady-state dynamics including ultrasensitivity and bistability were commonly found in responsive models as expected. The values of steady-state statistics *r_EA_*, *r_SU_* and *r_BI_* grouped by 36 initial concentration sets were listed in Table 2.

In contrast to steady-state dynamics, temporal dynamics including transient activation and oscillation were found in only few models (125 in 32,906).

Detailed analysis about the relationships between dynamics and model parameters will be described in Discussions.

## Discussion

### 4.1 Previous models of the MAPK cascade

Before discussing results of the generic model presented in this work, we first summarize the previous system-specific models listed in Table 1, on which the generic model have been based.

#### 4.1.1 Brief summaries of previous models

Model 1 was an integrated model including both the epidermal growth factor (EGF) signal pathway involving two receptors (EGFR and ErbB4) and the MAPK cascade. The model mapped the MAPK network of Chinese hamster ovary (CHO) cells to reveal the relationship between the network topology (feedbacks) and the network response. The result indicated that the model including a positive feedback from active MAPK to B-Raf (MAPKKK activated by ErbB4) and an inhibitory link from active B-Raf to active Raf-1 (MAPKKK activated by EGFR) could best fit the experiment data [28].

Model 2 mapped a network in Xenopus oocyte. The upstream pathway was simplified as a single input node. Some of the parameters were measured by experiments and the others estimated. The model predicted that the three-tier arrangement of the MAPK cascade could result in an ultrasensitive response. It was confirmed by further experiments in Xenopus oocyte [29].

Model 3 had been adjusted from Model 2 by adding a negative feedback from active MAPK to MAPKKK. Model 3 was not meant to map a specific biological system. The corresponding analysis indicated that a negative feedback coupled with ultrasensitivity could lead to oscillation [17].

Model 4 and Model 5 investigated the effects of scaffold proteins. The models were comprised of the core cascade like Model 2 [30].

Model 6 was an integrated model including the EGF signal pathway and the MAPK cascade of HeLa cells. The parameters have been trained with experimental data. Model 6 was built to predict the differential effects of surface and internalized EGF receptors [31].

Models 7–12 included only the last tier of the MAPK cascade, namely, from MAPKK to MAPK. These models revealed necessary conditions for bistability in dual-phosphorylation cycles, such as MAPK activation [15].

Model 13 was an integrated model of PC12 cells. Beside the MAPK cascade, this model also included two upstream signal pathways, namely, the EGF pathway and the NGF (neural growth factor) pathway. The model parameters were obtained by training using experimental data. The results of this model indicated that the distinct responses of the MAPK cascade to two input signals (EGF and NGF) were related to the different feedbacks from activated MAPK to the two upstream signal pathways [16].

#### 4.1.2 Possible dynamic behaviors displayed by previous models

After comparing 13 previous models in Table 1, possible relationships between dynamic behaviors and the network topologies can be summarized as below.

Ultrasensitivity was displayed by Models 2, 3, 4, 5 and 13, irrespective of the presence or absence of the upstream signal pathways and the feedback loops in the models. This suggests that these structural features are not absolutely necessary for ultrasensitivity. Model 2 indicated that the high degree of ultrasensitivity depended critically upon the total concentration of MAPKK and the two-collision mechanism of the dual phosphorylation reactions. In addition, Models 4 and 11 indicated that including the effects of scaffold proteins could change the ultrasensitivity of the MAPK activation by affecting the phosphorylation mechanisms of MAPKK and MAPK. It is also noteworthy that in different models, the ratios between [MAPKK]_total_ and [MAPK]_total_ were similar (∼1.0), while the absolute values of the respective quantities were significantly different.

Bistability of the MAPK pathway was investigated in Models 7–12, which contained only the dual-phosphorylation cycle of MAPK by MAPKK. The emergence of bistability was found to be caused by substrate saturation of the first phosphorylation step as well as the competitive inhibition of the second phosphorylation step by the substrate of the first step.

Transient activation of MAPK was predicted by Models 1 and 13. It was suggested that this type of dynamics was dependent on the upstream dynamics of respective adaptor proteins. However, comparisons of these two models with Model 6 suggested this may not be the only factor. Model 6 also included upstream EGF pathways but transient ERK activation was not found. The major difference between Model 6 and Models 1 and 13 lies in MAPK-mediated feedbacks. Both Models 1 and 13 contained this feedback to the upstream adaptor proteins, but there was no such feedback in Model 6. It is thus valuable to ask whether transient activation actually depends on the MAPK-mediated feedbacks to upstream pathways.

Sustained oscillation occurred only in Model 3. Model 3 was constructed by incorporating a MAPK-mediated negative feedback into Model 2. This feedback was considered to cause the oscillation.

### 4.2 Dynamics of the generic model

#### 4.2.1 Model responsiveness

Table 2 shows that the ratio of responsive models is critically determined by the ratios of pairs of total-concentration, including [MAPKK]_total_ over [MAPK]_total_ and substrates over phosphatases ([MAPKK]_total_ over [M2KP]_total_ and [MAPK]_total_ over [MKP]_total_). Particularly, the parameter groups in which [MAPKK]_total_≥[MAPK]_total_, or the total concentration of the phosphatases was smaller than those of their respective substrates (e.g. [M2KP]_total_<[MAPKK]_total_), are associated with significantly larger effective activating ratios.

**Figure 3:**
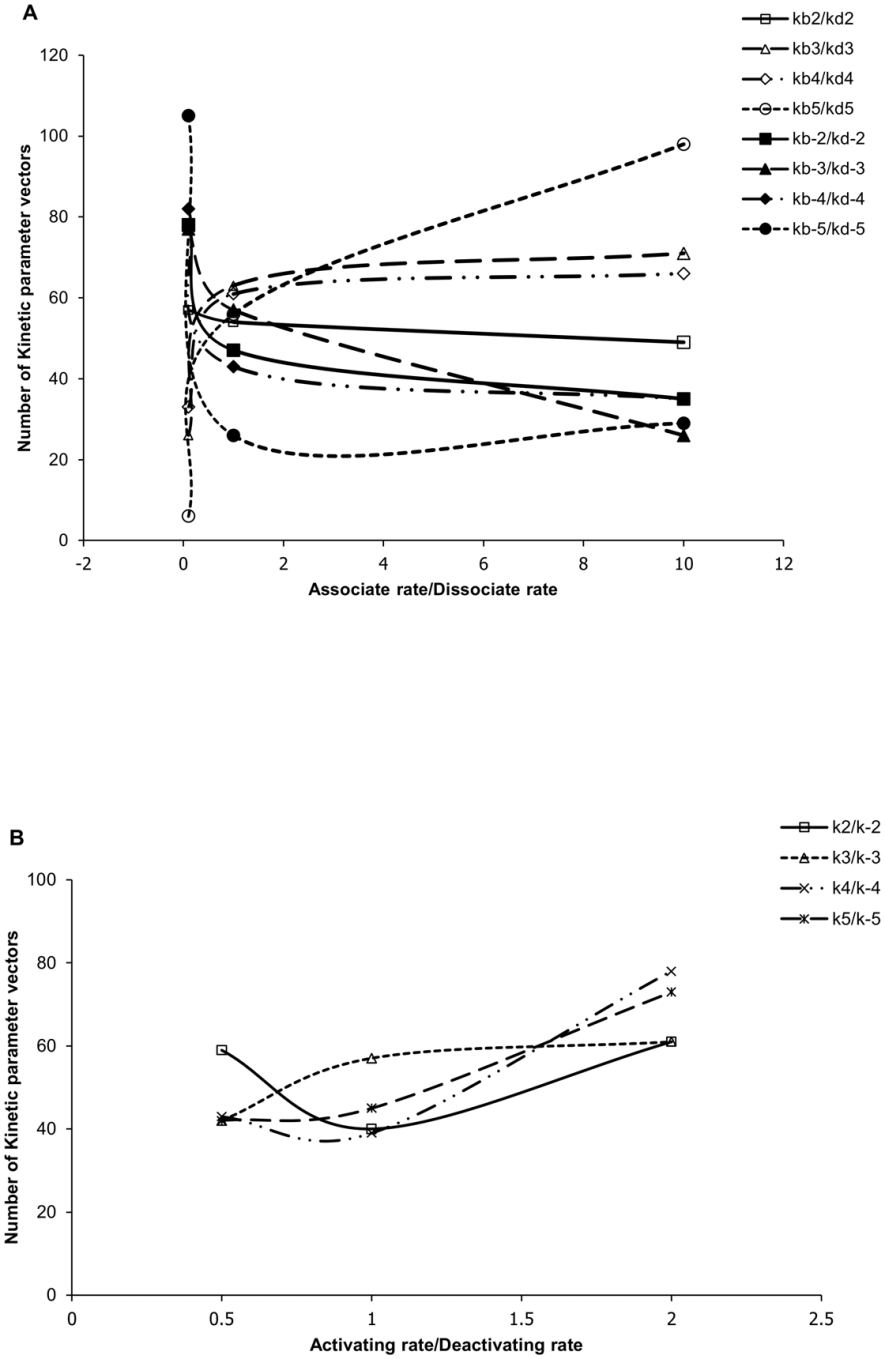
Number of kinetic parameter vectors (vertical axis) that, when combined with 36 concentration vectors, lead to responsive models in more than 30 cases. The total 160 kinetic parameter vectors are grouped by different ratios (horizontal axis) of (a) various association rates (kbN and kb-N) over the corresponding dissociation rates (kdN and kd-N), and (b) various activation rates (kN) over the corresponding deactivation rate (k-N).

Moreover, only 160 of 2000 kinetic parameter vectors can lead to responsive models in more than 30 cases when combined with the 36 concentration vectors. After comparing these kinetic parameter vectors, we found that the values of single kinetic parameters showed little effects, but the ratio of association over dissociation rates (e.g. kb5/kd5 in List S1, List S2 and List S3 of supplemental materials) as well as the ratio of phosphorylation over dephosphorylation rates (e.g. k2/k-2 in List S1, List S2 and List S3 of supplemental materials) significantly affected the responsive-model-inducing ability of a kinetic parameter vector (Figure 3).

The above results suggested that the MAPK cascade can be effectively blocked (i.e., made non-responsive) through increasing the amount of respective phosphatase.

#### 4.2.2 Ultrasensitivity

Table 2 shows that the ratio of ultrasensitive models strongly depends on the initial concentrations. A larger [MAPKK]_total_/[MAPK]_total_ ratio leads to larger *r_SU_*. This result means that with a fixed total concentration of MAPK, there is a positive correlation between the total amount of MAPKK and the ultrasensitivity of the network. This agrees with conclusions from previous Model 2 in Table 1, which suggested that the Hill coefficient of the overall response curve was positively correlated with the total amount of MAPKK.

Table 2 additionally suggests that a larger [MAPKK]_total_/[M2KP]_total_ ratio and a smaller [MAPK]_total_/[MKP]_total_ ratio lead to a larger *r_SU_* (e.g. concentration vector 02 versus concentration vector 03 in Table 2). Since a larger [MAPKK]_total_/[M2KP]_total_ ratio means more kinase (pp-MAPKK) of MAPK while smaller [MAPK]_total_/[MKP]_total_ ratio means more phosphatase of MAPK, this result reflects the correlation between ultrasensitivity and competitive binding of different enzymes with the same substrate.

In order to learn more about the relationship between [MAPKK]_total_ and ultrasensitivity, we drew *Gradient* versus [MAPKK]_total_ curves by scanning the values of [MAPKK]_total_ while fixing other parameters. To avoid scanning for all sets of parameters, we divided the parameter sets into three groups according to their respective *Gradient*. The first group was composed of parameter vectors having *Gradient* between (1.0, 10.0), representing “low ultrasensitivity”; the second group was composed of parameter vectors having *Gradient* between (10.0, 100.0), representing “medium ultrasensitivity”; the last group was composed of parameter vectors having *Gradient* larger than 100.0, representing “high ultrasensitivity”. We randomly chose 10 parameter sets from each group for the [MAPKK]_total_-scanning experiment. Consistent positive correlations between [MAPKK]_total_ and *Gradients* have been found in all of the three groups (Table 4).

**Table 4:** Correlation coefficients between [MAPKK]_total_ and *Gradient*/Signal Range.

*Gradient* a			Signal Range^b^		
Low^c^	Medium^c^	High^c^	Low^c^	Medium^c^	High^c^
0.99^d^	0.98	0.84	−0.55	−0.23	−0.52
0.99	0.99	0.99	−0.79	0.70	−0.58
0.99	0.97	0.61	0.19	−0.40	0.43
0.98	0.98	0.83	0.38	0.61	0.75
0.96	0.99	0.63	0.96	0.42	0.97
0.99	0.99	0.69	−0.63	0.40	0.95
1.00	0.97	0.96	−0.68	0.12	−0.75
0.99	0.90	0.97	−0.73	0.64	−0.64
0.96	0.92	0.89	−0.79	−0.36	0.26
0.97	0.87	0.98	0.40	0.03	0.25

a *Gradient* is an indicator for ultrasensitivity (see text).

b Signal range from the beginning to the end (maximum) of MAPKK activation.

c Sample sets grouped by *Gradients.* **Low**: samples having *Gradients* between (1.0, 10.0), representing “low ultrasensitivity”; **Medium**: samples having *Gradients* between (10.0, 100.0), representing “medium ultrasensitivity”; **High**: samples having *Gradients* larger than 100.0, representing “high ultrasensitivity”.

d Correlation coefficients between [MAPKK]_total_ (total concentration of MAPKK) and *Gradient* or Signal range.

We also found that with other parameters fixed, the signal ranges from the beginning to the end (maximum) of MAPKK activation were not correlated with [MAPKK]_total_ (Table 4), implying that the signal range was more or less invariant with respect to changes in [MAPKK]_total_. Therefore, with increasing [MAPKK]_total_, the amount of increase in pp-MAPKK upon a given increase in the input stimulus will become larger, leading to larger increase in the final output, which is the MAPK activity. In other words, when the concentration of pp-MAPKK needed for maximum activation of MAPK is determined by other parameters, a larger [MAPKK]_total_ results in a smaller signal difference between the beginning and the maximum of MAPK activation, leading to a larger *Gradient* or stronger overall ultrasensitivity of the network.

**Figure 4:**
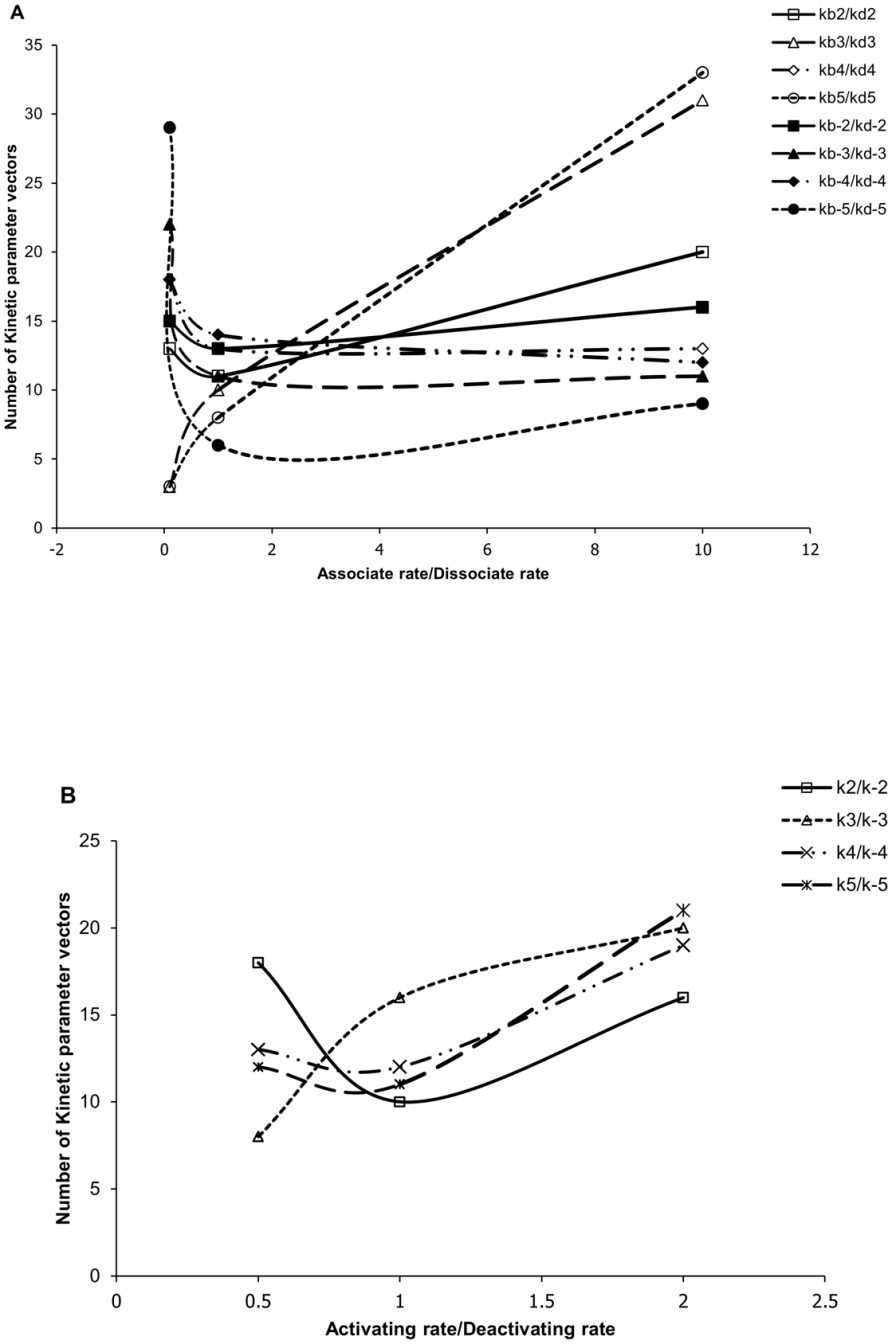
Number of kinetic parameter vectors (vertical axis) that lead to large *Gradient* (*Gradient*>400) for MAPK activation (see text). As in Figure 3, the numbers are shown for different ratios (horizontal axis) of (a) various association rates (kbN and kb-N) over the respective dissociation rates (kdN and kd-N), and (b) various activation rates (kN) over the respective deactivation rate (k-N).

Similar to the results for responsive models, the ratio of kinetic parameters associated with the second phosphorylation steps of MAPKK and MAPK (kb3/kd3, kb-3/kd-3, k3/k-3, kb5/kd5, kb-5/kd-5 and k5/k-5 in List S1, List S2 and List S3 of supplemental materials) showed very strong correlations with ultrasensitivity (Figure 4). The models in which the substrates have higher affinity to kinases (larger kb3/kd3 and larger kb5/kd5) and lower affinity to phosphatases (smaller kb-3/kd-3 and smaller kb-5/kd-5) are in general associated with larger *Gradient* or equivalently, stronger ultrasensitivity.

Irrespective of the presence or absence of feedback loops, over 50% models show significant ultrasensitivity (*Gradient*>1.0). This indicates that for the MAPK cascade, feedbacks are not dominating factors for ultrasensitivity. This result was also in agreement with a conclusion from the previous Model 2, which suggested that ultrasensitivity was dominated by the three-tier cascade arrangement rather than by the feedback loops.

#### 4.2.3 Bistability

Bistability was often considered as the result of positive feedbacks or dual-negative feedbacks [14], [32]. However, in our experiment, bistability was found in about 1/3 responsive models, irrespective of whether or what feedback loops the model contained. The ratios were also independent of feedbacks when concerning only absolutely bistable models.

Further analyses revealed the role of enzyme-substrate competitive binding in producing bistability. Since both MAPKK and MAPK have two phosphorylation sites sharing one kinase and one phosphatase, competitive binding may take place when two substrates bind to the same enzyme (e.g. MAPK and p-MAPK to pp-MAPKK), or when two enzymes bind to the same substrate (e.g. pp-MAPKK and MKP to p-MAPK). When competition for binding partners occurs, the steady state concentrations may become dependent on the initial concentrations, resulting in bistability. This feedback-independent mechanism has been discussed in [15] with simple models that included only the MAPK activating tier of the MAPK core cascade.

**Figure 5:**
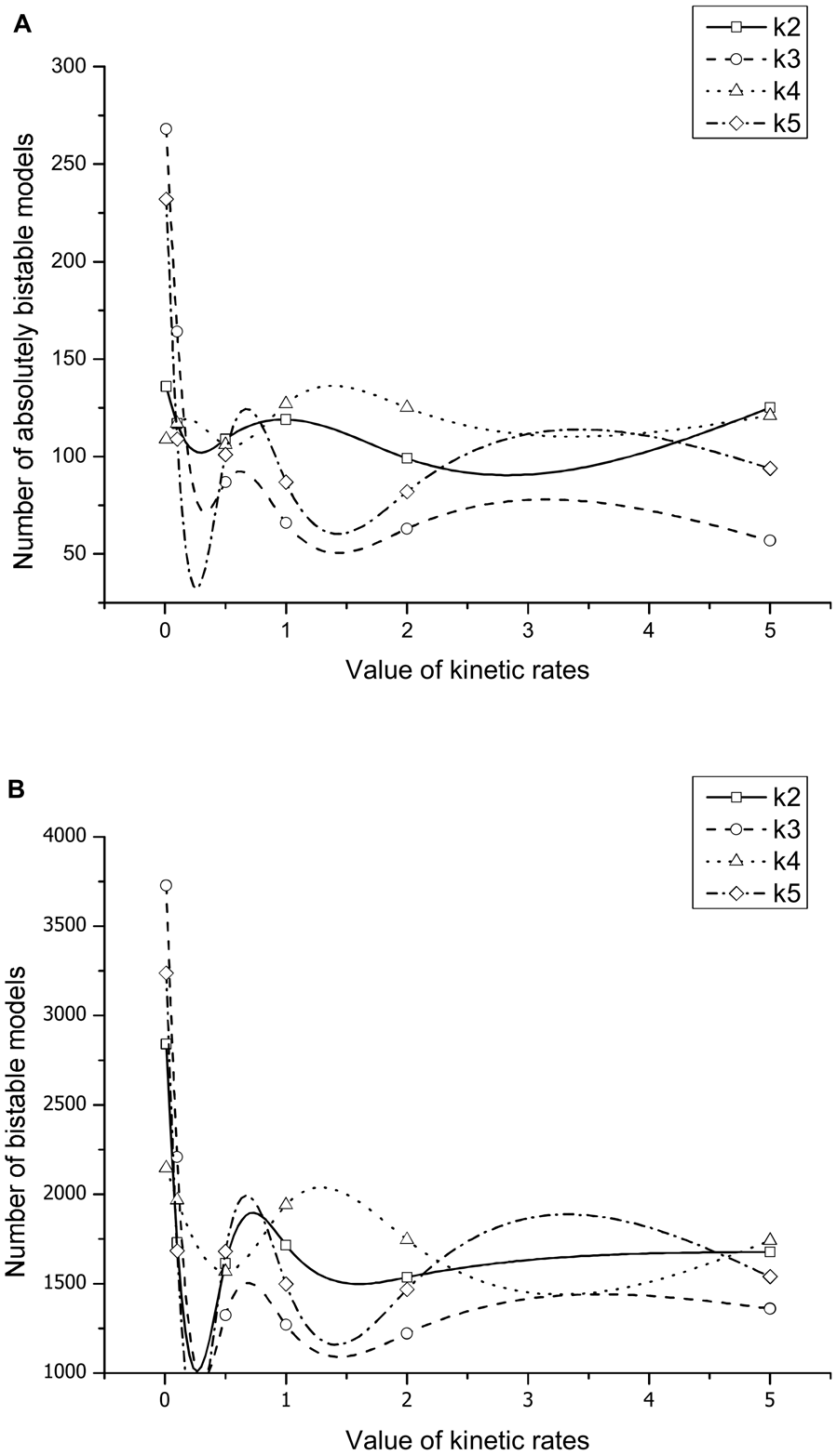
The numbers of bistable models obtained for different phosphorylation rates (horizontal axis). (a) Absolutely bistable models (*Bistability*>9.5); (b) Bistable models (*Bistability*>1.5).

Our analyses also revealed a qualitative correlation between the likelihood of obtaining absolutely bistable models and the phosphorylation rates of the second phosphorylation sites of MAPKK and MAPK (k3 and k5 in our model). Absolute bistability emerged easier in models with smaller k3 or k5 (Figure 5A). Similar results were found when concerning not only the absolutely bistable but also the remaining bistable models, except that the effects of smaller k2 (phosphorylation rate of the first phosphorylation site of MAPKK) also become significant (Figure 5B). The rate constant k5 determines the consumption rate of the intermediate product p-MAPK, while both k2 and k3 affect the concentration of MAPK kinase pp-MAPKK. Smaller k2, k3 or k5 would lead to increased p-MAPK and decreased pp-MAPKK, which eventually resulted in more intensive competitions for binding. Thus the numeric results support the above theory that bistability in the MAPK core cascade can be mainly caused by competitive binding.

We note that this cause of bistability is different from commonly discussed causes involving positive feedbacks. Besides our results here, it has been noted previously that oscillation or bistability could emerge for specific parameters of the MAPK cascade model without feedback loops [33]. In addition, Models 7–12 also indicated that particular parameter combinations coupled with competitive inhibition could lead to bistability in a simple dual-phosphorylation structure [15]. Therefore, positive feedback was in general not a necessary condition for bistability in MAPK activation. This conclusion, however, does not exclude that positive feedbacks, when present in particular MAPK systems, can cause bistable responses in MAPK cascade.

#### 4.2.4 Temporal dynamics

In our simulations, transient activation or oscillation was found in few models (125 in 32,906). In addition, all of the 125 models exhibiting transient activation and/or oscillation contained a negative feedback. Thus given the general topology of the MAPK core cascade, the presence of such temporal properties relies first on special topological features of the network, and then on specific parameter combinations.

As can be expected from the essentiality of negative feedbacks in generating non-trivial temporal dynamics, the total number of models exhibiting transient activation or oscillation increased from 125 to 398 when the effect of the negative feedback was strengthened relatively by reducing the strength of the input signal from 10.0 to 1.0 (however, with the decreased input strength, the number of models yielding effective MAPK activation decreased from 32,906 to 29,337). Apparently, the strength of the input signal is determined by upstream pathways. Therefore, although upstream pathways are not determinative on the steady-state dynamics of MAPK core cascade, it may play roles in determining the transient or temporal response of MAPK activation in a cascade containing negative feedbacks, through regulating the strength of the stimulus.

## Conclusions

In order to obtain an integrated view of the relationship between dynamics and architecture of the MAPK network, we have constructed a generic model of the MAPK cascade by comparing 13 previous MAPK models from 7 references, with the help of a reaction-based database. Systematic exploration of the parameter space, including total protein concentrations and kinetic constants, have been carried out to shed light onto the causal relationships between these quantitative properties of this ubiquitous network block and its different types of qualitative dynamics behaviors.

The generic MAPK cascade model can successfully reproduce reported steady-state dynamics (ultrasensitivity and bistability) and the temporal dynamics (transient activation and oscillation) observed for the MAPK pathways in different real systems. The results indicate that the complex dynamics of the MAPK pathways are mainly determined by the structure and the parameters contained in the three-tiered core cascade rather than by upstream pathways. This conclusion is in agreement with a previous work applying multi-parametric global sensitivity analysis to an integrated, system-specific MAPK network including both an upstream EGF signal pathway and the core cascade consisting of Ras and Raf (MAPKKK), MEK (MAPKK), and ERK (MAPK) [34]. In other words, the MAPK core cascade motif is sufficiently versatile, being capable of delivering a variety of qualitative dynamics required by different cellular signal transduction tasks through tunable parameters. This may be one of the reasons why the same MAPK core cascade are found widely in nature as the central blocks in different cell signaling pathways, some of them performing very different functions.

In modulating the steady-state dynamics of the core cascade, the MAPKK seems to play the most important role. The ratio between [MAPKK]_total_ and [M2KP]_total_ was found to be the most important factor to determine whether the cascade can yield effective response, i.e., MAPK activation, upon induction by a strong-enough upstream signal. In addition, it is the total amount of MAPKK that determines the ultrasensitivity of the response with respect to the stimulus. Moreover, competitive complex formation during dual phosphorylation of MAPKK can induce bistability in the absence of any apparent feedbacks.

It is possible that the central role of MAPKK in regulating the overall dynamics may give the MAPK core cascade some important advantages as a modular block in complicated cellular networks. Since MAPKK was the intermediate component of the MAPK core cascade, it may receive less interference from outside modules than the other two components. In other words, the determining role of MAPKK may make the MAPK cascade dynamics modular, being susceptible to regulations by specific signals but robust against unwanted external influences, for example, variations in the upstream network.

In the above analyses, we found the reaction-based database proposed in this work could indeed facilitate both model comparisons and the construct of generic models. We consider an integrated reaction-based database to be a useful tool in biological modeling to address questions which could be better understood through generic models pulling out from models focusing on specific systems rather than through the system-specific models themselves. Such questions may include the complex interplay and causal relations between topology, concentration constraints, kinetic rates and dynamics of biological networks.

## Supporting Information

List S1 **The biochemical reactions involved in the generic MAPK cascade model.**
(DOC)

List S2 **The mathematical equations of the generic MAPK cascade model for simulation.**
(DOC)

List S3 **The parameter sampling method used in the article.**
(DOC)

Table S1 **Definitions of major elements of reaction-based database.**
(DOC)

Text S1 **Data structure and unified format of reaction-based database.**
(DOC)
