# Supplementary material for: Random Parameter Sampling of a Generic Three-Tier MAPK Cascade Model Reveals Major Factors Affecting Its Versatile Dynamics
Source: PLoS One. 2013 Jan 24;8(1):e54441. doi: 10.1371/journal.pone.0054441 (PMC3554771; doi:10.1371/journal.pone.0054441)
Supplement: List S1 — The biochemical reactions involved in the generic MAPK cascade model. (DOC) [file pone.0054441.s001.doc]

#### List S1. Reactions.
