# Supplementary material for: Random Parameter Sampling of a Generic Three-Tier MAPK Cascade Model Reveals Major Factors Affecting Its Versatile Dynamics
Source: PLoS One. 2013 Jan 24;8(1):e54441. doi: 10.1371/journal.pone.0054441 (PMC3554771; doi:10.1371/journal.pone.0054441)
Supplement: List S2 — The mathematical equations of the generic MAPK cascade model for simulation. (DOC) [file pone.0054441.s002.doc]

**List S2. Equations.**

$$\begin{matrix} v_{1}=k_{1}[\text{Signal}][\text{MAPKKK}] \\ v_{21}=k_{b2}[\text{MAPKK}][\text{p-MAPKKK}]-k_{d2}[\text{MAPKK:p-MAPKKK}] \\ v_{22}=k_{2}[\text{MAPKK:p-MAPKKK}] \\ v_{31}=k_{b3}[\text{p-MAPKK}][\text{p-MAPKKK}]-k_{d3}[\text{p-MAPKK:p-MAPKKK}] \\ v_{32}=k_{3}[\text{p-MAPKK:p-MAPKKK}] \\ v_{41}=k_{b4}[\text{MAPK}][\text{pp-MAPKK}]-k_{d4}[\text{MAPK:pp-MAPKK}] \\ v_{42}=k_{4}[\text{MAPK:pp-MAPKK}] \\ v_{51}=k_{b5}[\text{p-MAPK}][\text{pp-MAPKK}]-k_{d5}[\text{p-MAPK:pp-MAPKK}] \\ v_{52}=k_{5}[\text{p-MAPK:pp-MAPKK}] \end{matrix}$$

$$\begin{matrix} v_{-1}=k_{-1}[\text{p-MAPKKK}] \\ v_{-21}=k_{b-2}[\text{p-MAPKK}][\text{M2KP}]-k_{d-2}[\text{p-MAPKK:M2KP}] \\ v_{-22}=k_{-2}[\text{p-MAPKK:M2KP}] \\ v_{-31}=k_{b-3}[\text{pp-MAPKK}][\text{M2KP}]-k_{d-3}[\text{pp-MAPKK:M2KP}] \\ v_{-32}=k_{-3}[\text{pp-MAPKK:M2KP}] \\ v_{-41}=k_{b-4}[\text{p-MAPK}][\text{MKP}]-k_{d-4}[\text{p-MAPK:MKP}] \\ v_{-42}=k_{-4}[\text{p-MAPK:MKP}] \\ v_{-51}=k_{b-5}[\text{pp-MAPK}][\text{MKP}]-k_{d-5}[\text{pp-MAPK:MKP}] \\ v_{-52}=k_{-5}[\text{pp-MAPK:MKP}] \end{matrix}$$

$$\begin{matrix} v_{a}=w_{a}[\text{Inactive Signal}][\text{pp-MAPK}] \\ v_{b}=w_{b}[\text{Signal}][\text{pp-MAPK}] \end{matrix}$$

$$\begin{matrix} \frac{d[\text{MAPKKK}]}{dt}=-v_{1}+v_{-1} \\ \frac{d[\text{p-MAPKKK}]}{dt}=v_{1}-v_{-1}-v_{21}+v_{22}-v_{31}+v_{32} \\ \frac{d[\text{MAPKK}]}{dt}=-v_{21}+v_{-22} \\ \frac{d[\text{MAPKK:p-MAPKKK}]}{dt}=v_{21}-v_{22} \\ \frac{d[\text{p-MAPKK}]}{dt}=v_{22}-v_{31}-v_{-21}+v_{-32} \\ \frac{d[\text{p-MAPKK:p-MAPKKK}]}{dt}=v_{31}-v_{32} \\ \frac{d[\text{pp-MAPKK}]}{dt}=v_{32}-v_{41}+v_{42}-v_{51}+v_{52}-v_{-31} \\ \frac{d[\text{MAPK}]}{dt}=-v_{41}+v_{-42} \\ \frac{d[\text{MAPK:pp-MAPKK}]}{dt}=v_{41}-v_{42} \\ \frac{d[\text{p-MAPK}]}{dt}=v_{42}-v_{51}-v_{-41}+v_{-52} \\ \frac{d[\text{p-MAPK:pp-MAPKK}]}{dt}=v_{51}-v_{52} \\ \frac{d[\text{pp-MAPK}]}{dt}=v_{52}-v_{-51} \\ \frac{d[\text{M2KP}]}{dt}=-v_{-21}+v_{-22}-v_{-31}+v_{-32} \\ \frac{d[\text{p-MAPKK:M2KP}]}{dt}=v_{-21}-v_{-22} \\ \frac{d[\text{pp-MAPKK:M2KP}]}{dt}=v_{-31}-v_{-32} \\ \frac{d[\text{MKP}]}{dt}=-v_{41}+v_{-42}-v_{-51}+v_{-52} \\ \frac{d[\text{p-MAPK:MKP}]}{dt}=v_{-41}-v_{-42} \\ \frac{d[\text{pp-MAPK:MKP}]}{dt}=v_{-51}-v_{-52} \\ \frac{d[\text{Inactive Signal}]}{dt}=v_{b}-v_{a} \\ \frac{d[\text{Signal}]}{dt}=v_{a}-v_{b} \end{matrix}$$
