# Supplementary material for: Random Parameter Sampling of a Generic Three-Tier MAPK Cascade Model Reveals Major Factors Affecting Its Versatile Dynamics
Source: PLoS One. 2013 Jan 24;8(1):e54441. doi: 10.1371/journal.pone.0054441 (PMC3554771; doi:10.1371/journal.pone.0054441)
Supplement: List S3 — The parameter sampling method used in the article. (DOC) [file pone.0054441.s003.doc]

**List S3. Parameter sampling.**

$$\begin{matrix} K_{Ori}=1.0; \\ AmpK_{cat}=\{0.01;0.1;0.5;1.0;2.0;5.0\}; \\ AmpBD=\{0.1;1.0;10.0;100.0\}; \\ AmpK_{s}=\{0.01;0.1;1.0;5.0\}; \\ AmpW_{ab}=\{0.2;1.0;5.0\}. \end{matrix}$$

$$\begin{matrix} k_{1}=K_{Ori}; \\ k_{2}=K_{Ori}\times AmpK_{cat}(1+floor(rand*6));k_{3}=K_{Ori}\times AmpK_{cat}(1+floor(rand*6)); \\ k_{4}=K_{Ori}\times AmpK_{cat}(1+floor(rand*6));k_{5}=K_{Ori}\times AmpK_{cat}(1+floor(rand*6)); \\ k_{-1}=k_{1}; \\ k_{-2}=AmpK_{cat}(3+floor(rand*3))\times k_{2};k_{-3}=AmpK_{cat}(3+floor(rand*3))\times k_{3}; \\ k_{-4}=AmpK_{cat}(3+floor(rand*3))\times k_{4};k_{-5}=AmpK_{cat}(3+floor(rand*3))\times k_{5}; \\ \\ k_{b2}=K_{Ori}\times AmpBD(1+floor(rand*4));k_{d2}=AmpBD(1+floor(rand*3))\times k_{b2}; \\ k_{b3}=K_{Ori}\times AmpBD(1+floor(rand*4));k_{d3}=AmpBD(1+floor(rand*3))\times k_{b3}; \\ k_{b4}=K_{Ori}\times AmpBD(1+floor(rand*4));k_{d4}=AmpBD(1+floor(rand*3))\times k_{b4}; \\ k_{b5}=K_{Ori}\times AmpBD(1+floor(rand*4));k_{d5}=AmpBD(1+floor(rand*3))\times k_{b5}; \\ k_{b-2}=K_{Ori}\times AmpBD(1+floor(rand*4));k_{d-2}=AmpBD(1+floor(rand*3))\times k_{b-2}; \\ k_{b-3}=K_{Ori}\times AmpBD(1+floor(rand*4));k_{d-3}=AmpBD(1+floor(rand*3))\times k_{b-3}; \\ k_{b-4}=K_{ori}\times AmpBD(1+floor(rand*4));k_{d-4}=AmpBD(1+floor(rand*3))\times k_{b-4}; \\ k_{b-5}=K_{Ori}\times AmpBD(1+floor(rand*4));k_{d-5}=AmpBD(1+floor(rand*3))\times k_{b-5}; \\ \\ k_{s}=K_{Ori}\times AmpK_{s}(1+floor(rand*4));k_{-s}=k_{s}; \\ w_{a}=\left\{\begin{matrix} K_{Ori}\times AmpW_{ab}(1+floor(rand*3)),\text{ if positive feedback;} \\ \text{0, if others. } \end{matrix}\right. \\ w_{b}=\left\{\begin{matrix} K_{Ori}\times AmpW_{ab}(1+floor(rand*3)),\text{ if negative feedback;} \\ \text{0, if others. } \end{matrix}\right. \end{matrix}$$

$$\begin{matrix} C_{Ori}=1.0; \\ AmpC1=\{0.2;1.0;5.0\}; \\ AmpC2=\{0.1;1.0\}. \end{matrix}$$
