# Supplementary material for: Random Parameter Sampling of a Generic Three-Tier MAPK Cascade Model Reveals Major Factors Affecting Its Versatile Dynamics
Source: PLoS One. 2013 Jan 24;8(1):e54441. doi: 10.1371/journal.pone.0054441 (PMC3554771; doi:10.1371/journal.pone.0054441)
Supplement: Table S1 — Definitions of major elements of reaction-based database. (DOC) [file pone.0054441.s004.doc]

**Table S1**. Major elements of reaction-based database.

| **Tables** | **Elements** | **Description** | **Remarks** |
| --- | --- | --- | --- |
| Reactions | Model ID | The ID of father model |  |
|  | Sub ID | The ID of reactions | Begin from 1 in each model. |
|  | Bio Type | Biological type of reactions | Combination, Phosphorylation, and etc... |
|  | Sim Type | Simulation type of reactions | ODE, Boolean, and etc… |
|  | Reactant | List of reactants | Sub class of Species |
|  | Product | List of products | Sub class of Species |
|  | Enzyme | List of enzymes | Sub class of Species |
|  | Parameter | List of parameters | Sub class of Parameter |
|  | Rule | Mathematical rule | Unified symbol expression |
|  | Characteristic | Special properties for simulation | Non-delta, FixRi, and etc…Optional |
|  | Comments | Optional descriptions of reactions |  |
|  |  |  |  |
| Species | Model ID | The ID of father model |  |
|  | Sub ID | The ID of species | Begin from 1 in each model |
|  | Bio Types | Biological types of species | Protein, Enzyme, DNA, and etc… |
|  | Sim Type | Simulation type of species | Substance, Symbol, Pool, and etc… |
|  | System ID | System ID of enzymes | Optional |
|  | Nick Names | Nick names of species | Unified format |
|  | Initial Concentration | Initial concentrations of species | Exclude units |
|  | Threshold | Thresholds of species | Optional |
|  | Unit | Unit of initial concentration and threshold | Exclude time unit |
|  | Comments | Optional descriptions of species |  |
|  |  |  |  |
| Parameters | Value | Values of parameters | Exclude units |
|  | Unit | Unit of parameters | Time unit |
