# Supplementary material for: Random Parameter Sampling of a Generic Three-Tier MAPK Cascade Model Reveals Major Factors Affecting Its Versatile Dynamics
Source: PLoS One. 2013 Jan 24;8(1):e54441. doi: 10.1371/journal.pone.0054441 (PMC3554771; doi:10.1371/journal.pone.0054441)
Supplement: Text S1 — Data structure and unified format of reaction-based database. (DOC) [file pone.0054441.s005.doc]

**Text S1. Data structure and unified format of reaction-based database**

Each reaction is an individual data record of the table “Reactions”. The properties of reactants, products and enzymes are stored in the table “Species”. Reactions and Species are grouped by the element “Model ID”. The expressions (data formats) of reactions and species are unified. Same reactions (species) in different models can be found with same keywords.

The nicknames of species are unified by following definitions:

1. The basic name or abbreviation of specie should be consistent with STRING ([http://string-db.org](http://string.embl.de/)) if it is a real molecular. For example, ERK.
2. If the molecular is a member of a large family and named in the form “family name + number”, the number should abut on the family name. For example, Caspase3 but not Caspase-3.
3. The modification of molecular should be added in front of the basic name and linked with “-“. For example, pp-ERK but not ERK-pp.
4. The additional properties of the species, such as the activity and the position in cell, should be added in the end of the basic name, and linked with “_”. In addition, the active form of molecular can be marked with a star mark (*) abutting on the basic name. For example, p-TrkA_endo, PI3K* or PI3K_active.
5. Using ‘:’ to separate the components of a complex. For example, EGF:EGFR.
6. Polymers should be expressed as “(Monomer)Number”. For example, (EGF:EGFR)2.

The kinetic equations of reactions are unified by converting into symbolic equations. A symbol includes a letter and an abutting number. Letters represent different objects: **R** for Reactant, **U** for prodUct, **E** for Enzyme, and **P** for Parameter. Numbers abutting on the letters indicate the order or sub-IDs of the objects in the reaction. For example, **R1** means the first (**1**^st^) reactant (**R**) of a reaction. With this definition, kinetic equations of reactions of a same or similar kinetic type can be expressed as a same symbolic equation. For example, all of the first order kinetic equations can be expressed as a same symbolic equation “**P1*R1**”.
